# A Mediterranean Dietary Pattern Is Associated with Greater Participation in Physical Activity and Better Health-Related Quality of Life among Students and Professors at the Melilla Campus (University of Granada)

**DOI:** 10.3390/nu15183971

**Published:** 2023-09-14

**Authors:** María López-Olivares, Philip Sapp, Terrence M. Riley, Penny M. Kris-Etherton, Carmen Enrique-Mirón, Teresa Nestares, Kristin M. Davis

**Affiliations:** 1 Department of Nutrition and Food Science, Faculty of Health Sciences, Melilla Campus, University of Granada, C/Santander s/n, 52001 Melilla, Spain; 2 Department of Nutritional Sciences, Pennsylvania State University, University Park, PA 16802, USA; 3 HUM-613 Research Group, Department of Inorganic Chemistry, Faculty of Health Sciences, Melilla Campus, University of Granada, C/Santander s/n, 52001 Melilla, Spain; 4 Department of Physiology, Faculty of Pharmacy, University of Granada, 18071 Granada, Spain; 5 Biomedical Research Centre (CIBM), Institute of Nutrition and Food Technology “José MataixVerdú” (INYTA), University of Granada, 18071 Granada, Spain; 6 Department of Psychology, Wayne State University, Detroit, MI 48202, USA

**Keywords:** Mediterranean diet, hrQOL, lifestyle, university, physical activity, affect

## Abstract

The objective of this study was to assess Mediterranean diet (MD) scores (i.e., alignment with a MD pattern) among students and professors, in addition to assessing how adherence to the MD was associated with other lifestyle behaviors. A cross-sectional observational study was conducted with a sample of 127 university professors and 272 students of the Melilla Campus at the University of Granada (Spain). Students were more physically active than professors (mean difference = 1058 METs, *p* < 0.001) and reported lower negative affect (NA; mean difference = −1.70, *p* < 0.001) whereas professors reported nominally better perceived mental health. For the total sample, the physical health component (β = 0.03, *p* = 0.03) and physical activity (β = 0.0001, *p* = 0.01) were significantly associated with higher MD scores. Health behaviors, including MD scores and physical activity, were suboptimal among both students and professors. The results suggest that a dietary pattern reflective of the MD is positively associated with both physical and mental health outcomes among students and professors, though the direction of the associations remains to be clarified.

## 1. Introduction

Diet quality is closely related to the risk of cardiovascular disease (CVD), which is the leading cause of death globally [1]. Dietary risks, such as low intake of fruits or high intake of processed meats, are the second leading modifiable risk factor for CVD across 195 countries [1]. Poor diet quality may also increase the risk of mental health disorders, including depression and anxiety, although the directions of these associations remain unclear [2,3,4,5]. Hence, poor diet quality is a daunting problem worldwide and an important preventable risk factor for both chronic diseases and mental health disorders. Beyond a poor-quality diet, other behavioral and psychological factors contribute to CVD risk, including low physical activity [1,6] and negative affect [7]. According to the World Health Organization (WHO), adults should participate in 150 to 300 min of moderate, or 75 to 150 min of vigorous intensity physical activity each week [8]; lower levels of physical activity increase the risk of both CVD [9] and depression [10]. Importantly, these risk factors tend to cluster and interact with each other [11,12]. Thus, improvements in diet quality may improve other health domains, and vice versa.

The Mediterranean diet (MD) is a high-quality dietary pattern with widely recognized health benefits [13,14,15], including reduced CVD risk [16,17]. The MD is comprised primarily of unprocessed foods, including vegetables, fruits, whole grains, legumes, nuts, beans, fish, and olive oil, and is limited in red meat and dairy products [18], which closely aligns with the diet and nutrition guidelines promoted by the World Health Organization [19]. Importantly, a dietary pattern that aligns with the MD may benefit both mental and physical health [20]. Herein the term “adherence” refers to alignment with the MD pattern, rather than the degree to which individuals follow a prescribed dietary pattern. The physical health benefits of MD, such as reduced risk of CVD and metabolic syndrome, have been studied extensively. Evidence also suggests that maintaining a MD pattern may reduce the risk of certain cancers [21,22,23], though differences in how MD is operationalized complicate the interpretation of these results [3]. Studies investigating the potential mental health benefits of MD adherence, while limited, report promising findings [19]. For example, several studies have reported negative associations between MD adherence and depressive symptoms [24,25,26]; negative associations between MD adherence and negative affect, a predominant depressive symptom, have also been reported [27]. The MD is also associated with better health-related quality of life (hrQOL), a measure of both perceived mental and physical health, among adults with and without osteoarthritis [28,29]. These limited, yet positive associations between a MD and mental health warrant further investigation.

Incoming college students experience many lifestyle changes, including decreases in diet quality and physical activity [30,31,32]. These health behaviors tend to remain poor throughout students’ time in college [33,34,35,36]. Interestingly, college students from countries where the MD is the traditional diet tend to score poorly on MD scoring tools and consume a diet characterized by excessive alcohol, fat, and sugar, and low in fruits, vegetables, and pulses [37]. Transitioning to college is also associated with negative effects on mental health. One in three college students experience clinically significant mental health issues [38], and rates of mental health problems among college students have increased [39]. Despite this, little research has examined the health behaviors and mental health of university professors. One study reported that university professors do not meet guidelines for MD adherence or physical activity [40], while another study found that both students and professors have generally poor health behaviors, including low consumption of fruits and vegetables and inadequate physical activity, as well as high stress levels [41].

A healthy diet, physical activity, and mental health are important factors influencing CVD morbidity and mortality. Understanding the current lifestyle practices of college students and faculty may help inform the development of targeted interventions to improve mental and physical health, particularly among members of the higher education community. Thus, the objective of this study was to assess whether the dietary pattern of students and professors at the Melilla Campus of the University of Granada aligns with a MD. In addition, we assessed the association between alignment with a MD and other lifestyle behaviors and mental health factors, including physical activity, positive and negative affect, and health-related quality of life (hrQOL).

## 2. Materials and Methods

### 2.1. Study Design, Participants and Procedures

A cross-sectional observational study was conducted on a sample of university professors (i.e., assistant and full professors who were full-time faculty) and students at the University of Granada, Melilla Campus (Spain). Students in any academic year were potentially eligible for participation. All eligible professors on the campus were contacted. Students were recruited via emails and in-class announcements. Participants were not compensated. Participants were selected using incidental non-probability sampling. All questionnaires were completed in person. The data were collected between October 2019 and February 2020. This study was conducted in accordance with the directives established by the Declaration of Helsinki. The study protocol was approved by the University of Granada Institutional Review Board. All participants provided written informed consent.

### 2.2. Methods

Sociodemographic data were collected via a self-administered questionnaire. To assess adherence to the MD, we used the Mediterranean Diet Adherence Screener (MEDAS). This validated 14-point questionnaire was used in PREDIMED [42], a large multicenter nutrition intervention trial, that evaluated the effects of a Mediterranean diet versus a low-fat diet on cardiovascular disease events [43,44]. Higher MEDAS scores indicate diets that more closely align with the MD; scores of ≥9 are reflective of a MD pattern. The Positive and Negative Affect Schedule (PANAS) questionnaire was used to assess positive and negative affect. The PANAS questionnaire [45] consists of 20 items; of these, 10 measure the positive effect (PA, e.g., cheerful or thrilled) and 10 items measure negative effect (NA, e.g., tense or stressed). Scores for each subscale can range from 0 to 50. Higher scores on the PA subscale reflect greater PA, whereas for the NA subscale, they reflect greater NA.

Health-related quality of life (hrQOL) was assessed using the Short Form Health Survey 36 version 2 (SF36). The SF36 consists of 36 items, assessing eight dimensions: physical functioning (PF), physical role functioning (PR), body pain (BP), general health (GH), vitality (VT), social functioning (SF), emotional role functioning (ER), and mental health (MH). This questionnaire provides an overall score representing hrQOL and allows for the calculation of two components: physical health component (PF + PR + BP + GH) and mental health component (ER + SF + MH + VT) [46].

Physical activity was assessed using the International Physical Activity Questionnaire, short version (IPAQ) [47], in which participants reported the amount of time spent engaging in moderate activity, vigorous activity, walking, and sitting over the past week. Weekly activity was recorded in Metabolic Equivalent of Task (METs) per minute and week. To score these data, the Guidelines for Data Processing and Analysis of the IPAQ [47] were followed.

Data were collected among enrolled students, assistants, and full-time faculty professors at the University of Grenada in Melilla, Spain. Melilla is a Spanish city located in North Africa, on the shores of the Mediterranean Sea, and is influenced by Mediterranean, European, and African cuisines. In addition, Melilla is a city that is supplied with fresh foods such as fish, meat, fruits, and vegetables in alignment with a Mediterranean dietary pattern. With mild winters, Melilla is well suited for a healthy lifestyle year-round, including regular physical activity. The Melilla campus has outdoor sports facilities where students can engage in physical activity, as well as a dining hall where they can enjoy a healthy diet.

### 2.3. Analyses

All analyses were completed with IBM SPSS Statistics for Windows, Version 24.0 (International Business Machines Corporation (IBM), Armonk, NY, USA). Figures were made in RStudio (version 4.2.2) [48] using ggplot2 (version 3.4.1) [49]. Significance was accepted at *p* < 0.05. Prior to beginning analyses, Kolmogorov–Smirnov tests were used to evaluate the normality of the data. If not normally distributed, the data were log-transformed as needed.

The representative sample size was calculated using the formula described by Spiegel and Stephens [50]. Representative sample size was estimated based on the total number of professors and students who were at the Melilla Campus in the 2019/2020 academic year. For professors, we estimated that 100 were needed; for students, we estimated the representative sample size to be 212. We enrolled approximately 25% more than the required sample size. Thus, a sample of 127 professors and 272 students were enrolled in the study.

To determine whether students and professors differed significantly in their overall hrQOL, SF-36 scores were compared via a *t*-test. Similarly, between-group differences in the physical health component, mental health component, PA, NA, physical activity, MEDAS scores, and each component of the MD were also compared using *t*-tests.

Associations between MEDAS scores and hrQOL, PA, NA, and physical activity were assessed using multiple regression with ordinary squares estimation. In the regression model, standardized and non-standardized regression coefficients were calculated.

## 3. Results

A total of 399 participants completed the study, of which 127 were professors and 272 were students. On average, the students were aged 21 ± 4 (standard deviation) and professors were 47 ± 11 years old. The sample predominantly identified as female (60.40%). Most participants reported being in the health sciences (49.70%). Table 1 summarizes participant characteristics. Mean MEDAS adherence scores were 8.50 ± 1.90 in the total sample, 8.59 ± 1.98 among students, and 8.29 ± 1.77 among professors. MEDAS score adherence data are presented in Table 2.

Students were more physically active than professors (mean difference = 1058 METs, *p* < 0.001) and had lower NA (mean difference = −1.70, *p* < 0.001). For the mental health component of hrQOL, the difference between students and professors approached, but did not reach, significance (mean difference = 1.47, *p* = 0.053), with professors reporting nominally better perceived mental health. MEDAS adherence scores for professors were nominally lower (8.29 ± 1.77) than students (8.59 ± 1.98), though the difference did not reach significance. For both the physical health component and NA, the professors’ scores were nominally better, (74.76 ± 7.78 and 18.47 ± 7.25, respectively), although not significantly different, than the students’ (73.09 ± 7.55 and 16.77 ± 6.07, respectively) (Table 3).

**Figure 1:**
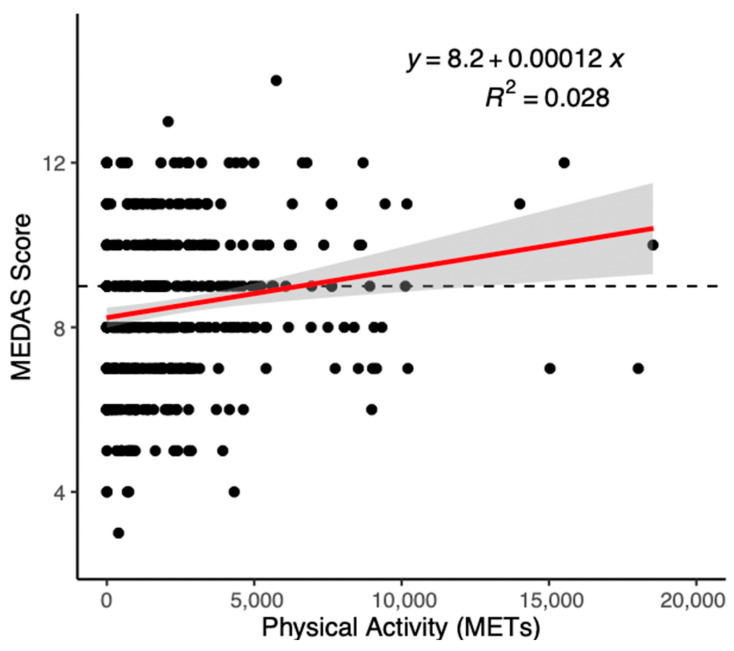
Scatter plot of the relationship between MEDAS score and physical activity. Note: MEDAS, Mediterranean Diet Adherence Screener; METs, Metabolic Equivalent of Task.

**Figure 2:**
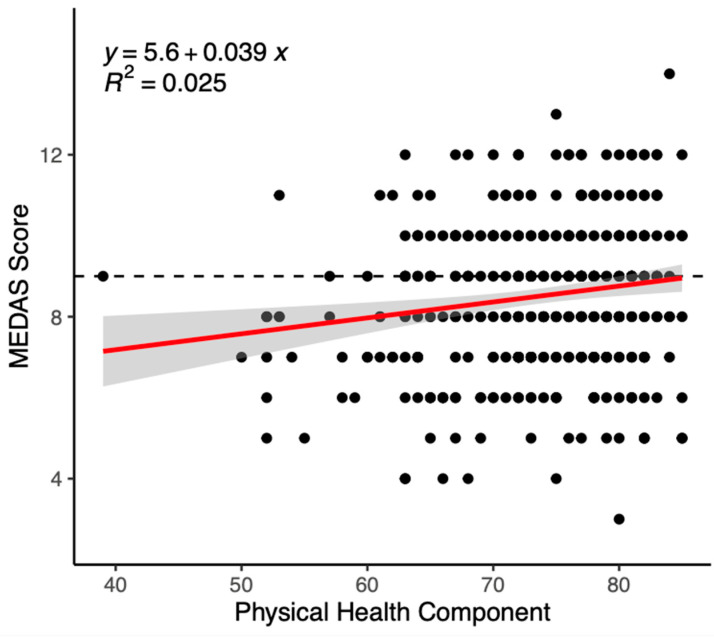
Scatter plot of the relationship between MEDAS score and the physical health component of hrQOL. Note: hrQOL, health-related quality of life; MEDAS, Mediterranean Diet Adherence Screener.

Regression results are presented in Table 4. For the total sample, the physical health component (β = 0.03, *p* = 0.03) and physical activity (β = 0.00, *p* = 0.01) were significantly positively associated with MEDAS scores. The association between MD adherence and PA approached, but did not reach, significance (β = 0.03, *p* = 0.05). Results are presented in Table 4, as well as in Figure 1 and Figure 2.

## 4. Discussion

The present cross-sectional study examined adherence to the MD in a sample of students and professors at the Melilla Campus of the University of Granada and tested whether MD scores were associated with health behaviors and mental health factors, including physical activity, PA, NA, and hrQOL. Students were more physically active and had lower NA compared to professors; however, MD adherence was similar across the two groups. Higher MD adherence scores were associated with better scores on the physical health component of hrQOL, and with higher physical activity scores. While college students are frequently a target population for research, studies of professors are less common. Additionally, few studies have compared the health behaviors and subjective health of university students and professors. A large body of research has found that lifestyle and mental health factors are suboptimal to disparately poor among college students, e.g., [34,51,52,53]. Interestingly, we found that students and professors did not significantly differ in most variables of interest, including MD score, perceived physical health, and positive affect. This study, therefore, provides evidence that these factors could be improved in both students and professors. Future research to develop lifestyle interventions in university settings will be important to improve the health and well-being of this population.

Students and professors differed significantly in two key areas: students reported significantly more physical activity than professors, as well as significantly lower NA. Physical activity generally declines with increasing age [54]; age-related differences between students and professors may therefore contribute to the observed difference in physical activity between the two subgroups. However, because neither students nor professors met physical activity guidelines, efforts should be made to increase physical activity levels overall. In contrast with our findings, past research has often reported that NA decreases with increasing age [55,56,57]. However, one study reported that older adults are more likely to experience increased NA in response to stress compared to younger adults [56]. The present study did not examine stress, and we are unable to determine whether stress was a factor in the observed results. More research examining the influence of stress among university students and professors is needed to better understand its role in affecting dynamics in the university setting.

Among students and professors in the present study, MEDAS scores were associated with significantly more physical activity. The direction of this association cannot be determined due to the cross-sectional study design. Still, this adds to a growing body of research suggesting that health behaviors tend to cluster. For example, evidence suggests that individuals with high-quality diets, including MD, tend to engage in more physical activity or less sedentary behavior [58,59,60], in line with the present findings. Similarly, MD adherence was significantly associated with the physical health component of the SF-36. This could suggest that either those who feel physically well are more able to adhere to a higher quality diet, and/or that consuming a higher quality diet leads to feeling better physically. Longitudinal studies are needed. Additionally, the present study observed a marginal association between PA and MD adherence (*p* = 0.054); while this association did not reach statistical significance, the effect size is similar to the association between MEDAS scores and the physical health component. Past research has yielded mixed results regarding the association between PA and health behaviors, including diet [61]. Previous research has found that MD adherence is associated with higher PA [62], while some studies show no association [63]. Additional prospective longitudinal studies are needed to clarify the association between the MD pattern and PA.

The finding that participants had low MD adherence scores is consistent with existing research reporting low MD adherence in Spain [64] and declining MD adherence both throughout the Mediterranean region and globally [65,66,67]. A longitudinal study published in 2010 reported that MD adherence in Spain declined significantly from 1987 through 1997, stabilized briefly, and then actually improved significantly from 2001 through 2005 [68]. However, it is unclear if this improvement has continued. More recent work has reported low MD adherence in Spain, with Southeastern Spain having the lowest adherence [64], while another study reported good adherence to the MD among university students in Italy [69]. Nonetheless, the bulk of the evidence appears to demonstrate low levels of adherence to the MD, especially among younger populations, in European and Mediterranean nations, including Spain [64], Greece [70], Portugal [71], and Italy [59], as well as Croatia [72], Albania [73], Lithuania [74], and the Western Balkans broadly [75]. It is important to note, however, that there is substantial variation in the way that MD is defined across nations and individual studies [76], making comparisons across studies challenging.

There is evidence to suggest that the trend away from the traditional MD in the Mediterranean region is strongest among younger populations [77]. We observed no difference in diet scores between professors and students, suggesting that the trend away from the MD is a concern for both younger and middle-aged adults. At least one other previous study also reported no differences in MD scores across age groups in the Mediterranean region [78]. It is possible that other factors, such as socioeconomic status, may account for the observed differences in MD adherence across the lifespan. Socioeconomic status is consistently associated with diet quality and MD adherence, e.g., [71,79,80]. Higher levels of education, specifically, are also associated with better adherence to the MD [59,78], making the present finding of poor MD adherence in university professors particularly troubling. Further, evidence suggests that economic decline may be a driving factor in declining MD adherence, both in the Mediterranean population and globally [67,80]. Research investigating effective methods for improving MD adherence, especially for populations of low socioeconomic status, is needed.

While university students are common participants in scientific studies, research regarding the health and health behaviors of university faculty is lacking. University faculty are a highly educated group and are therefore likely to be aware of the importance of maintaining good health behaviors. Despite this, our results suggest that health behaviors are suboptimal in this population. Studies examining barriers to maintaining positive health behaviors among faculty could be useful for fostering an environment that encourages a healthy diet and lifestyle as part of the university experience. Additionally, given the poor MD adherence and low levels of physical activity reported, interventions targeting the health behaviors of university professors are warranted. Similarly, a large body of evidence suggests that university students have particularly poor health behaviors [59,64,70,71,72,73,74,75], in agreement with the present study. For example, studies conducted at the Universities of Alicante, Albacete, Cuenca, Navarra, Madrid, Granada, and Huelva all reported that adherence to the MD among students was low [81,82,83,84,85]. However, evidence identifying effective interventions is lacking. Research regarding policies and practices that could facilitate healthy behaviors for both professors and students could lead to substantial health benefits for both groups. Universities should consider developing policies and allocating resources to enable better health behaviors in both students and professors.

The present study is limited by the use of a cross-sectional design, which precludes the ability to draw conclusions about the directionality of the associations. High-quality longitudinal studies examining the relationship between a MD, physical activity, and physical and mental health are needed. The study is further limited by the inclusion of only self-reported measures; no biometric data were collected. Future research involving biomarkers (e.g., cholesterol) and anthropometric measures (e.g., body mass index) would be valuable. Such work would help clarify whether the present results regarding the association between MD and health in students and professors hold when using more objective measures of health, rather than self-reported perceived health-related quality of life. Finally, the study is limited by the use of a convenience sample. While this sample may be largely representative of students and faculty at the University of Melilla, our results may not generalize to other universities or settings.

## 5. Conclusions

Health behaviors, including adherence to the MD quality scores and physical activity, were suboptimal among both students and professors. Efforts to facilitate positive health behaviors are warranted. A MD pattern was related to both perceived physical health and physical activity. Promoting interventions that improve MD diet quality scores would therefore be expected to improve both physical and mental health outcomes among students and professors.

## Figures and Tables

**Table 1:** Baseline characteristics of participants (*n* = 399).

	Total (*n* = 399)		Students (*n* = 272)		Professors (*n* = 127)	
	Mean ± SD		Mean ± SD		Mean ± SD	
Age	29.34 ± 14.15		20.97 ± 3.71		47.28 ± 11.36	
	Frequency	%	Frequency	%	Frequency	%
Gender						
Males	158	39.6	96	35.5	62	48.8
Females	241	60.4	176	64.5	65	51.2
Study						
Health Sciences	198	49.7	169	61.9	29	22.8
Education Sciences	143	35.8	103	38.1	40	31.5
Social Sciences	31	7.8	0	0	31	24.4
Administration	27	7.6	0	0	27	21.3
Place of origin						
Melilla	228	57.1	148	54.2	80	63.0
Other Spanish territory	171	42.9	124	45.8	47	37.0

**Table 2:** Frequency of MD criteria achieved by university professors and students as determined by MEDAS tool.

	Total (*n* = 399)		Students (*n* = 272)		Professors (*n* = 127)	
MD Recommendations	Frequency	(%)	Frequency	(%)	Frequency	(%)
Use of olive oil as the main source of fat	383	96.0	260	95.6	123	96.9
Consumption of ≥4 spoons (20 mL) of olive per day	343	86.0	233	85.7	110	86.6
Consumption of ≥2 portions (100 g) of vegetables per day	193	48.4	156	57.4	37	29.1
Consumption of ≥3 portions (pieces) of fruit per day	139	34.8	95	34.9	44	34.6
Consumption of <1 portion (100–150 g) of red meat, burgers, sausages, or cold meat per day	270	67.7	173	63.6	97	76.4
Consumption of <1 portion (12 g) of butter, margarine, or cream per day	286	72.0	184	67.7	103	81.1
Consumption of <1 portion of soft or sugary drinks per day	275	68.9	174	64.0	101	79.5
Consumption of ≥1 portion of wine	41	10.3	14	5.1	27	21.3
Consumption of ≥3 portions (150 g) of legumes per week	164	41.1	133	48.9	31	24.4
Consumption of ≥3 portions (100–150 g) of fish or seafood per week	197	49.4	135	49.6	62	48.8
Consumption of <3 portions of industrial pastry per week	251	62.9	167	61.4	84	66.1
Consumption of ≥3 portions (30 g) of nuts per week	241	60.4	165	60.7	76	59.8
Preference of chicken meat, turkey, or rabbit to beef, pork, burgers, or sausages (100–150 g)	332	83.2	241	88.6	91	71.7
Consumption of ≥2 portions of whole grains (with pasta, rice, or sauté meals) per week	282	70.7	216	79.4	66	52.0
	Mean	SD	Mean	SD	Mean	SD
MEDAS Score	8.50	1.90	8.59	1.98	8.29	1.77

**Table 3:** State of health including lifestyle factors (i.e., physical activity, Mediterranean diet), health-related quality of life, and emotional well-being of the total cohort and subgroups (values are means ± standard deviations).

	Students (*n* = 272)	Professors (*n* = 127)	*p*
	Mean ± SD	Mean ± SD	
hrQOL			
Physical health component	73.09 ± 7.55	74.76 ± 7.78	0.238
Mental health component	53.24 ± 7.48	54.71 ± 8.09	0.053
Emotional state			
PA	31.60 ± 8.07	33.91 ± 7.76	0.574
NA	16.77 ± 6.07	18.47 ± 7.25	<0.001 ***
Physical activity			
METs	2794.76 ± 2975.76	1736.77 ± 2504.03	<0.001 ***
MEDAS score	8.59 ± 1.98	8.29 ± 1.77	0.195

hrQOL, health-related quality of life; PA, positive affect; NA, negative affect; METs, Metabolic Equivalent of Task; MEDAS, Mediterranean Diet Adherence Screener. Between-group differences were analyzed using the Mann–Whitney U. *p*-value: *** <0.001.

**Table 4:** Results of the regression model testing the relationship between MEDAS score and baseline characteristics, hrQOL components, and physical activity in the total sample (N = 399).

	MEDAS Score			
Variable	B (SE)	95% CI (Lower, Upper)	β	*p*
Intercept	6.78 (1.17)	4.49, 9.06		<0.001 *
Sex (Ref = M)	−0.35 (0.20)	−0.75, 0.04	−0.09	0.08
Subgroup ^a^	−0.22 (0.21)	−0.64, 0.19	−0.05	0.29
Physical health component	0.03 (0.01)	0.00, 0.06	0.13	0.03 *
Mental health component	−0.01 (0.02)	−0.04, 0.03	−0.03	0.65
PA	0.03 (0.01)	0.00, 0.05	0.11	0.05
NA	−0.03 (0.02)	−0.06, 0.00	−0.09	0.12
Physical activity (METs)	0.0001 (0.00003)	0.00, 0.00	0.129	0.01 *

Note: ^a^ Subgroup is comprised of professors and students; MEDAS, Mediterranean Diet Adherence Screener; hrQOL, health-related quality of life; METs, Metabolic Equivalent of Task; SE, standard error; β, standardized coefficient; * *p* < 0.05.

## Data Availability

No new data were created or analyzed in this study. Data sharing is not applicable to this article.

## References

[1] Roth G.A., Mensah G.A., Johnson C.O., Addolorato G., Ammirati E., Baddour L.M., Barengo N.C., Beaton A.Z., Benjamin E.J., Benziger C.P. (2020). Global Burden of Cardiovascular Diseases and Risk Factors, 1990–2019: Update from the GBD 2019 Study. J. Am. Coll. Cardiol..

[2] Bremner J.D., Moazzami K., Wittbrodt M.T., Nye J.A., Lima B.B., Gillespie C.F., Rapaport M.H., Pearce B.D., Shah A.J., Vaccarino V. (2020). Diet, Stress and Mental Health. Nutrients.

[3] Godos J., Currenti W., Angelino D., Mena P., Castellano S., Caraci F., Galvano F., Del Rio D., Ferri R., Grosso G. (2020). Diet and Mental Health: Review of the Recent Updates on Molecular Mechanisms. Antioxidants.

[4] Collins S., Dash S., Allender S., Jacka F., Hoare E. (2022). Diet and Mental Health During Emerging Adulthood: A Systematic Review. Emerg. Adulthood.

[5] Solomou S., Logue J., Reilly S., Perez-Algorta G. (2023). A systematic review of the association of diet quality with the mental health of university students: Implications in health education practice. Health Educ. Res..

[6] Han J., Yin H., Wang J. (2020). Examining the Relationships Between Job Characteristics, Emotional Regulation and University Teachers’ Well-Being: The Mediation of Emotional Regulation. Front. Psychol..

[7] Nabi R.L., Clark S. (2008). Exploring the Limits of Social Cognitive Theory: Why Negatively Reinforced Behaviors on TV May Be Modeled Anyway. J. Commun..

[8] Bull F.C., Al-Ansari S.S., Biddle S., Borodulin K., Buman M.P., Cardon G., Carty C., Chaput J.-P., Chastin S., Chou R. (2020). World Health Organization 2020 guidelines on physical activity and sedentary behaviour. Br. J. Sports Med..

[9] Ahmed H.M., Blaha M.J., Nasir K., Rivera J.J., Blumenthal R.S. (2012). Effects of physical activity on cardiovascular disease. Am. J. Cardiol..

[10] Kandola A., Ashdown-Franks G., Hendrikse J., Sabiston C.M., Stubbs B. (2019). Physical activity and depression: Towards understanding the antidepressant mechanisms of physical activity. Neurosci. Biobehav. Rev..

[11] Schultchen D., Reichenberger J., Mittl T., Weh T.R.M., Smyth J.M., Blechert J., Pollatos O. (2019). Bidirectional relationship of stress and affect with physical activity and healthy eating. Br. J. Health Psychol..

[12] Kris-Etherton P.M., Sapp P.A., Riley T.M., Davis K.M., Hart T., Lawler O. (2022). The Dynamic Interplay of Healthy Lifestyle Behaviors for Cardiovascular Health. Curr. Atheroscler. Rep..

[13] Guasch-Ferré M., Willett W.C. (2021). The Mediterranean diet and health: A comprehensive overview. J. Intern. Med..

[14] Kastorini C.-M., Panagiotakos D.B., Chrysohoou C., Georgousopoulou E., Pitaraki E., Puddu P.E., Tousoulis D., Stefanadis C., Pitsavos C. (2016). Metabolic syndrome, adherence to the Mediterranean diet and 10-year cardiovascular disease incidence: The ATTICA study. Atherosclerosis.

[15] Sofi F., Cesari F., Abbate R., Gensini G.F., Casini A. (2008). Adherence to Mediterranean diet and health status: Meta-analysis. BMJ.

[16] Rosato V., Temple N.J., La Vecchia C., Castellan G., Tavani A., Guercio V. (2019). Mediterranean diet and cardiovascular disease: A systematic review and meta-analysis of observational studies. Eur. J. Nutr..

[17] Grosso G., Marventano S., Yang J., Micek A., Pajak A., Scalfi L., Galvano F., Kales S.N. (2017). A comprehensive meta-analysis on evidence of Mediterranean diet and cardiovascular disease: Are individual components equal?. Crit. Rev. Food Sci. Nutr..

[18] Davis C., Bryan J., Hodgson J., Murphy K. (2015). Definition of the Mediterranean Diet: A Literature Review. Nutrients.

[19] Nishida C., Uauy R., Kumanyika S., Shetty P. (2004). The joint WHO/FAO expert consultation on diet, nutrition and the prevention of chronic diseases: Process, product and policy implications. Public Health Nutr..

[20] Ventriglio A., Sancassiani F., Contu M.P., Latorre M., Di Slavatore M., Fornaro M., Bhugra D. (2020). Mediterranean Diet and its Benefits on Health and Mental Health: A Literature Review. Clin. Pr. Epidemiol. Ment. Health.

[21] Mentella M.C., Scaldaferri F., Ricci C., Gasbarrini A., Miggiano G.A.D. (2019). Cancer and Mediterranean diet: A review. Nutrients.

[22] Barak Y., Fridman D. (2017). Impact of Mediterranean diet on cancer: Focused literature review. Cancer Genom. Proteom..

[23] D’alessandro A., De Pergola G., Silvestris F. (2016). Mediterranean Diet and cancer risk: An open issue. Int. J. Food Sci. Nutr..

[24] Parletta N., Zarnowiecki D., Cho J., Wilson A., Bogomolova S., Villani A., Itsiopoulos C., Niyonsenga T., Blunden S., Meyer B. (2019). A Mediterranean-style dietary intervention supplemented with fish oil improves diet quality and mental health in people with depression: A randomized controlled trial (HELFIMED). Nutr. Neurosci..

[25] Jacka F.N., O’Neil A., Opie R., Itsiopoulos C., Cotton S., Mohebbi M., Castle D., Dash S., Mihalopoulos C., Chatterton M.L. (2017). A randomised controlled trial of dietary improvement for adults with major depression (the «SMILES» trial). BMC Med..

[26] Sánchez-Villegas A., Martínez-González M.A., Estruch R., Salas-Salvadó J., Corella D., Covas M.I., Arós F., Romaguera D., Gómez-Gracia E., Lapetra J. (2013). Mediterranean dietary pattern and depression: The PREDIMED randomized trial. BMC Med..

[27] Esteban-Gonzalo L., Turner A.I., Torres S.J., Esteban-Cornejo I., Castro-Piñero J., Delgado-Alfonso Á., Marcos A., Gómez-Martínez S., Veiga L. (2019). Diet quality and well-being in children and adolescents: The UP&DOWN longitudinal study. Br. J. Nutr..

[28] Muñoz M.A., Fíto M., Marrugat J., Covas M.I., Schröder H., REGICOR and HERMES Investigators (2009). Adherence to the Mediterranean diet is associated with better mental and physical health. Br. J. Nutr..

[29] Veronese N., Stubbs B., Noale M., Solmi M., Luchini C., Maggi S. (2016). Adherence to the Mediterranean diet is associated with better quality of life: Data from the Osteoarthritis Initiative. Am. J. Clin. Nutr..

[30] El Ansari W., Stock C., John J., Deeny P., Phillips C., Snelgrove S., Adetunji H., Hu X., Parke S., Stoate M. (2011). Health promoting behaviours and lifestyle characteristics of students at seven universities in the UK. Cent. Eur. J. Public. Health.

[31] Lee I.M., Shiroma E.J., Lobelo F., Puska P., Blair S.N., Katzmarzyk P.T., Lancet Physical Activity Series Working Group (2012). Effect of physical inactivity on major non-communicable diseases worldwide: An analysis of burden of disease and life expectancy. Lancet.

[32] Vadeboncoeur C., Townsend N., Foster C. (2015). A meta-analysis of weight gain in first year university students: Is freshman 15 a myth?. BMC Obes..

[33] Keating X.D., Guan J., Piñero J.C., Bridges D.M. (2005). A meta-analysis of college students’ physical activity behaviors. J. Am. Coll. Health.

[34] Small M., Bailey-Davis L., Morgan N., Maggs J. (2013). Changes in eating and physical activity behaviors across seven semesters of college: Living on or off campus matters. Health Educ. Behav..

[35] Ramón-Arbués E., Granada-López J.M., Martínez-Abadía B., Echániz-Serrano E., Antón-Solanas I., Jerue B.A. (2021). Factors Related to Diet Quality: A Cross-Sectional Study of 1055 University Students. Nutrients.

[36] Wilson O.W.A., Panza M.J., Evans M.B., Bopp M. (2021). A Scoping Review on College Student Physical Activity: How Do Researchers Measure Activity and Examine Inequities?. J. Phys. Act. Health.

[37] Hadjimbei E., Botsaris G., Gekas V., Panayiotou A.G. (2016). Adherence to the Mediterranean Diet and Lifestyle Characteristics of University Students in Cyprus: A Cross-Sectional Survey. J. Nutr. Metab..

[38] Auerbach R.P., Mortier P., Bruffaerts R., Alonso J., Benjet C., Cuijpers P., Demyttenaere K., Ebert D.D., Green J.G., Hasking P. (2018). WHO World Mental Health Surveys International College Student Project: Prevalence and distribution of mental disorders. J. Abnorm. Psychol..

[39] Xiao H., Carney D.M., Youn S.J., Janis R.A., Castonguay L.G., Hayes J.A., Locke B.D. (2017). Are we in crisis? National mental health and treatment trends in college counseling centers. Psychol. Serv..

[40] Egeda Manzanera J.M., Rodrigo Vega M. (2014). Adherencia a la Dieta Mediterránea en futuras maestras. Nutr. Hosp..

[41] Melnyk B.M., Hsieh A.P., Tan A., Gawlik K.S., Hacker E.D., Ferrell D., Simpson V., Burda C., Hagerty B., Scott L.D. (2021). The state of mental health and healthy lifestyle behaviors in nursing, medicine and health sciences faculty and students at Big 10 Universities with implications for action. J. Prof. Nurs..

[42] Martínez-González M.A., García-Arellano A., Toledo E., Bes-Rastrollo M., Bullo M., Corella D., Fito M., Ros E., Lamuela-Raventós R.M., Rekondo J. (2014). Obesity Indexes and Total Mortality among Elderly Subjects at High Cardiovascular Risk: The PREDIMED Study. PLoS ONE.

[43] Ros E. (2017). The PREDIMED study. Endocrinol. Diabetes Y Nutr..

[44] Guillem Saiz P., Yang Wang Y., Guillem Saiz J., Fernández V., Saiz Sánchez C. (2017). Estilos de vida, adherencia a la dieta mediterránea, características antropométricas en un colectivo de universitarios de ciencias de la salud. Rev. Esp. Nutr. Comunitaria.

[45] Watson D., Clark L.A., Tellegen A. (1988). Development and validation of brief measures of positive and negative affect: The PANAS scales. J. Personal. Soc. Psychol..

[46] Rodríguez-Romero B., Pita-Fernández S., Pertega Díaz S., Chouza-Insua M. (2013). Calidad de vida relacionada con la salud en trabajadoras del sector pesquero usando el cuestionario SF-36. Gac. Sanit..

[47] Mantilla Toloza S.C., Gómez Conesa A.A. (2007). El cuestionario internacional de actividad física: Un instrumento adecuado en el seguimiento de la actividad física poblacional. Rev. Iberoam. De Fisioter. Y Kinesiol..

[48] R Core Team (2022). R: A Language and Environment for Statistical Computing.

[49] Wickham H. (2009). ggplot2: Elegant Graphics for Data Analysis.

[50] Vargas D. Estadística. Serie Schaum 4ta edición Murray R. Spiegel.pdf (1). https://www.academia.edu/44609733/Estad%C3%ADstica_Serie_Schaum_4ta_edici%C3%B3n_Murray_R_Spiegel_pdf_1.

[51] Huang T.T.K., Harris K.J., Lee R.E., Nazir N., Born W., Kaur H. (2003). Assessing overweight, obesity, diet, and physical activity in college students. J. Am. Coll. Health.

[52] Pedrelli P., Nyer M., Yeung A., Zulauf C., Wilens T. (2015). College students: Mental health problems and treatment considerations. Acad. Psychiatry.

[53] Sheldon E., Simmonds-Buckley M., Bone C., Mascarenhas T., Chan N., Wincott M., Gleeson H., Sow K., Hind D., Barkham M. (2021). Prevalence and risk factors for mental health problems in university undergraduate students: A systematic review with meta-analysis. J. Affect. Disord..

[54] Stewart K.J. (2005). Physical activity and aging. Ann. N. Y. Acad. Sci..

[55] Shallcross A.J., Ford B.Q., Floerke V.A., Mauss I.B. (2013). Getting better with age: The relationship between age, acceptance, and negative affect. J. Pers. Soc. Psychol..

[56] Charles S.T., Reynolds C.A., Gatz M. (2001). Age-related differences and change in positive and negative affect over 23 years. J. Pers. Soc. Psychol..

[57] Thomsen D.K., Mehlsen M.Y., Viidik A., Sommerlund B., Zachariae R. (2005). Age and gender differences in negative affect--Is there a role for emotion regulation?. Personal. Individ. Differ..

[58] Berrigan D., Dodd K., Troiano R.P., Krebs-Smith S.M., Barbash R.B. (2003). Patterns of health behavior in U.S. adults. Prev. Med..

[59] Grosso G., Marventano S., Buscemi S., Scuderi A., Matalone M., Platania A., Giorgianni G., Rametta S., Nolfo F., Galvano F. (2013). Factors Associated with Adherence to the Mediterranean Diet among Adolescents Living in Sicily, Southern Italy. Nutrients.

[60] Olson J.S., Hummer R.A., Harris K.M. (2017). Gender and Health Behavior Clustering among U.S. Young Adults. Biodemography Soc. Biol..

[61] Steptoe A., Dockray S., Wardle J. (2009). Positive affect and psychobiological processes relevant to health. J. Pers..

[62] Holt M.E., Lee J.W., Morton K.R., Tonstad S. (2014). Mediterranean diet and emotion regulation. Med. J. Nutr. Metab..

[63] Moreno-Agostino D., Caballero F.F., Martín-María N., Tyrovolas S., López-García P., Rodríguez-Artalejo F., Haro J.M., Ayuso-Mateos J.L., Miret M. (2019). Mediterranean diet and wellbeing: Evidence from a nationwide survey. Psychol. Health.

[64] Abellán Alemán J., Zafrilla Rentero M.P., Montoro-García S., Mulero J., Pérez Garrido A., Leal M., Guerrero L., Ramos E., Ruilope L.M. (2016). Adherence to the “Mediterranean Diet” in Spain and Its Relationship with Cardiovascular Risk (DIMERICA Study). Nutrients.

[65] Vilarnau C., Stracker D.M., Funtikov A., da Silva R., Estruch R., Bach-Faig A. (2019). Worldwide adherence to Mediterranean Diet between 1960 and 2011. Eur. J. Clin. Nutr..

[66] Da Silva R., Bach-Faig A., Quintana B.R., Buckland G., de Almeida M.D.V., Serra-Majem L. (2009). Worldwide variation of adherence to the Mediterranean diet, in 1961–1965 and 2000–2003. Public Health Nutr..

[67] Obeid C.A., Gubbels J.S., Jaalouk D., Kremers S.P., Oenema A. (2022). Adherence to the Mediterranean diet among adults in Mediterranean countries: A systematic literature review. Eur. J. Nutr..

[68] Bach-Faig A., Fuentes-Bol C., Ramos D., Carrasco J.L., Roman B., Bertomeu I.F., Cristià E., Geleva D., Serra-Majem L. (2011). The Mediterranean diet in Spain: Adherence trends during the past two decades using the Mediterranean Adequacy Index. Public Health Nutr..

[69] Grillone L., Castriotta L., Antinolfi F., Righini M., Brusaferro S., Parpinel M. (2018). University students’ Mediterranean diet adherence in North East of Italy: A pilot study, 2018. Eur. J. Public Health.

[70] Martimianaki G., Eleni P., Elisavet V., Eleni M., Papatesta E., Trichopoulou A. (2014). Today’s Mediterranean Diet in Greece: Findings from the National Health and Nutrition Survey—HYDRIA (2013–2014). Nutrients.

[71] Mendonça N., Gregório M.J., Salvador C., Henriques A.R., Canhão H., Rodrigues A.M. (2022). Low adherence to the Mediterranean diet is associated with poor socioeconomic status and younger age: A cross-sectional analysis of the EpiDoC cohort. Nutrients.

[72] Šarac J., Havaš Auguštin D., Lovrić M., Stryeck S., Šunić I., Novokmet N., Missoni S. (2021). A generation shift in Mediterranean diet adherence and its association with biological markers and health in Dalmatia, Croatia. Nutrients.

[73] Llanaj E., Hanley-Cook G.T. (2021). Adherence to healthy and sustainable diets is not differentiated by cost, but rather source of foods among young adults in Albania. Br. J. Nutr..

[74] Mieziene B., Emeljanovas A., Novak D., Kawachi I. (2019). The relationship between social capital within its different contexts and adherence to a Mediterranean diet among Lithuanian adolescents. Nutrients.

[75] El Bilali H., Cardone G., Bottalico F., Palmisano G.O., Acquafredda A., Capone R. (2021). Mediterranean diet in the Western Balkans. Agrofor.

[76] Noah A., Truswell A.S. (2001). There are many Mediterranean diets. Asia Pac. J. Clin. Nutr..

[77] Tur J.A., Romaguera D., Pons A. (2023). Food Consumption Patterns in a Mediterranean Region. Ann. Nutr. Metab..

[78] Cavaliere A., De Marchi E., Banterle A. (2018). Exploring the Adherence to the Mediterranean Diet and Its Relationship with Individual Lifestyle: The Role of Healthy Behaviors, Pro-Environmental Behaviors, Income, and Education. Nutrients.

[79] Bonaccio M.B.R.M., Bes-Rastrollo M., De Gaetano G., Iacoviello L. (2016). Challenges to the Mediterranean diet at a time of economic crisis. Nutr. Metab. Cardiovasc. Dis..

[80] Merhout F., Doyle J. (2019). Socioeconomic status and diet quality in college students. J. Nutr. Educ. Behav..

[81] Cervera Burriel F., Serrano Urrea R., Vico García C., Milla Tobarra M., García Meseguer M.J. (2013). Hábitos alimentarios y evaluación nutricional en una población universitaria. Nutr. Hosp..

[82] Cobo-Cuenca A.I., Garrido-Miguel M., Soriano-Cano A., Ferri-Morales A., Martínez-Vizcaíno V., Martín-Espinosa N.M. (2019). Adherence to the Mediterranean Diet and Its Association with Body Composition and Physical Fitness in Spanish University Students. Nutrients.

[83] Durá Travé T., Castroviejo Gandarias A. (2011). Adherencia a la dieta mediterránea en la población universitaria. Nutr. Hosp..

[84] Ibáñez E.M., Ortega F.Z., Jiménez J.L.U., Valero G.G. (2021). Niveles de adherencia a la dieta mediterránea e inteligencia emocional en estudiantes del tercer ciclo de educación primaria de la provincia de Granada. Retos.

[85] López Nieves G., Sosa Cordobés E., Garrido Fernández A., Travé González G., García Padilla F.M. (2019). Hábitos, preferencias y habilidades culinarias de estudiantes de primer curso de la universidad de Huelva. Enfermería Glob..

